# A Question of Duty: Common Law Legal Issues Resulting from Physician Response to Unsolicited Patient Email Inquiries

**DOI:** 10.2196/jmir.2.3.e17

**Published:** 2000-09-18

**Authors:** Patricia C Kuszler

Patients have eagerly embraced the Internet and its email capacity to increase their knowledge and access to medical information. Along with access to countless patient support "chat rooms" and an ever-increasing volume of full text health and medical literature, the Internet also offers a virtually barrier-less opportunity to engage physicians in email dialogue. While this opportunity is seductive and cost-free to patients, physicians should exercise care and wariness in their email exchanges with patients - especially if the patient is unknown to them.

This issue of the *Journal of Medical Internet Research* features an interesting study illustrating the willingness of an astoundingly high percentage of anesthesiologists to enter into a dialogue with unknown patients [1]. The study, which looks at the quality and quantity of anesthesiologist responses to a patient problem presented through email communication, generated a 54% response rate. Of these, 83% of the responses were assessed as friendly in tone and 41% went so far as to suggest a diagnosis to the inquiring email patient. The study reproduces the results of an earlier study with similar methodology [2], demonstrating a surprising naiveté on the part of well-meaning physicians.

This brief commentary will summarize the common law legal risks of responding and offering advice or diagnostic suggestions to patients over the Internet. It will focus primarily on the concept of duty and how it has evolved in the United States. (Medical negligence cases in the United States are state law causes of action. Thus there are jurisdictional differences from state to state. However, the concept of duty as discussed in this article is well-settled law, and the consensus in virtually all U.S. jurisdictions.)

The risks in providing email medical advice flow from the inadvertent birth of a physician-patient relationship and a resulting duty and responsibility to the patient. Once there is a cognizable physician-patient relationship, the physician has a duty to provide care and advice that is consistent with the applicable standard of care. The standard of care varies somewhat from jurisdiction to jurisdiction. However, it generally requires that the physician conform to the standard of a reasonably prudent practitioner practicing under similar circumstances. The standard typically does take into account specialty status. Medical negligence is defined as a breach of the standard of care resulting in the patient suffering a harm with quantifiable damages.

In order for a physician-patient relationship to be formed, there must be an assent by the physician to see or counsel the patient, although this may be indirect. In the common law, this agreement is referred to as an implicit contract. In order for a duty relationship to have been formed, the content of the interaction must include some evaluation, even if only a rudimentary one, by the physician as to the patient's complaint. Finally, the patient must rely upon the physician's determination, however preliminary that evaluation might have been.

## Lessons learnt from cases related to telephone communication

Valuable lessons can be learned from the case law that has accumulated with respect to telephone communications between physicians and patients. Like the Internet, the telephone allows patients to access physicians directly and provides an instrument by which an implicit contract to provide care could be initiated. In the context of telephone communication, a patient call to a physician requesting and scheduling an appointment for the future does not necessarily result in the formation of a contract and the creation of a physician-patient relationship [see Textbox 1]. The physician may decline to accept the patient by refusing to schedule an appointment, or may condition acceptance on certain criteria or requirements [see Textbox 2]. One could argue that this situation approximates that in which a prospective patient seeks to consult or query the physician by email. In the email context, the physician could refuse to participate by simply not responding, issuing a reply declining to engage in an interaction, and/or suggesting the patient consult their own physician for medical advice and diagnosis.

However, if in the course of making an appointment, the patient is given some indication over the telephone that the physician has indeed agreed to provide care for the instant episode of illness, reasonably assumes that care is forthcoming, and relies upon that assumption by ceasing further efforts to obtain care for the condition, then a relationship giving rise to a duty will have been formed. The content of the interaction must show that the physician has undertaken to provide care for the patient for this episode of illness. Once the physician has "undertaken" to provide care, he is compelled to see the care through to its natural conclusion [3].

**A patient call to a physician requesting and scheduling an appointment for the future does not necessarily result in the formation of a contract and the creation of a physician-patient relationship**

For example, in Weaver v. University of Michigan Board of Regents, 201 Mich. App. 239, 506 N.W. 2d 264 (1993) a medical center neurosurgeon had cared for a child with hydrocephalus during her infancy. Parent later received follow-up care from another neurosurgeon nearer their home. Several years later, when the child developed vision complications, the father sought a second opinion from the medical center neurosurgeon. In obtaining the appointment, he volunteered the information that the child had been seen by the local neurosurgeon who did not consider the condition to be emergent. An appointment was obtained one week hence. The medical center neurosurgeon correctly diagnosed increased intracranial pressure and recommended emergency surgery. Although the surgery was a success, the child had already suffered permanent damage to her vision. The father sued all the caregivers, including the medical center neurosurgeon. The latter successfully argued that at the time the child suffered the harm, there had been no physician-patient relationship with the medical center and its physicians.

**The physician may decline to accept the patient by refusing to schedule an appointment, or may condition acceptance on certain criteria or requirements**

For example, in Childers v. Frye, 201 NC 41, 156 SE 744 (1931), the physician asked to see a victim from a motor vehicle accident observed that the patient appeared intoxicated and declined to provide care for the patient.

Even if the physician provides a tentative offer to provide care, the patient may fail to fulfill his role in forming the implicit contract. For example, in Miller v. Sulllivan, 214 A.D. 2d 822, 625 N.Y.S. 2d 102 (1995), a dentist experiencing back pain, shortness of breath, and other symptoms called a physician friend and related his complaint. The physician urged the dentist to come to the physician's office immediately for evaluation. The dentist essentially disregarded this advice, finished seeing his scheduled patients and then proceeded to the physician's office, where moments after arrival, he suffered a cardiac arrest. Here, the court held that the physician-patient relationship had not been formed because the plaintiff had essentially disregarded the very preliminary advice offered over the telephone.

For example, in Lyons v. Grether [4], a patient requested an appointment with a specialist physician for care of a specific complaint related to the physician's particular area of practice. Relying upon the assurance that he would see her, she arrived at his office with her guide dog and child in tow. The physician refused to see her unless she left her dog outside. Concerned for the safety and security of the dog, she insisted the dog remain. Thereupon, the physician reneged on his agreement to see her and evicted her from the office. The court held that because the plaintiff's appointment had been granted for the care of a specific ailment within the specialty expertise of the physician, it indicated the formation of a physician-patient relationship and a duty for the physician to perform that service: "Whether a physician-patient relationship is created is a question of fact, turning upon a determination whether the patient entrusted his treatment to the physician and the physician accepted the case."

Similarly, in Bienz v. Central Suffolk Hospital [5], the court held that a telephone conversation in which a physician provided advice to the patient, which the patient relied upon, constituted a physician-patient relationship and gave rise to a duty on the part of the physician [6].

However, if the patient failed to rely upon the advice provided over the telephone, the mere fact that the physician conversed with the patient on the telephone and listened to a recital of symptoms is not sufficient to form a physician-patient relationship. For example, in Clanton v. Von Haam [7], a patient with severe back pain called a physician she had previously seen for other complaints. The physician listened to her complaints and refused to see her that evening but agreed to see her in the morning, if her pain persisted. The court held that although the patient might have relied upon this advice, in this case the plaintiff had not relied upon the defendant physician.

## How does this apply to email?

Applying these principles to an email interaction such as that posed in Dr. Oyston's study, the fictional patient made a request - albeit somewhat veiled - for evaluation of the medical problem. The patient sought the physician's advice without any enticement or invitation from the physician. Several of the anesthesiologist respondents entered into an email dialogue, asked additional clinical questions, and 41% of them suggested a diagnosis. Providing a diagnosis could easily be assumed to be an undertaking to provide care to the patient. For example, if the physician has suggested a specific diagnosis and even discussed potential treatments, the patient may well rely upon this diagnosis and advice. If the diagnosis is in error or falsely reassuring and, as a result, the patient sustains harm, the patient would have a viable negligence action against the email physician. Assume, for instance, that the email anesthesiologist reassured the inquiring patient that her prior anesthesia complication was likely a one-in-a-million fluke and irrelevant to future care. The patient might well dismiss the concern from her mind and not even mention it to the next provider, who happens to be a surgeon, consulted to perform an elective surgery on the patient. The patient again responds abnormally to succinylcholine and, as a result, suffers a neurologic deficit. Having relied upon the email advice, the patient will almost certainly include the email anesthesiologist on the defendant list. The other defendants will welcome their email colleague as his negligent advice serves to decrease their potential liability.

## Recommendations for physicians confronted with unsolicited email

Dr. Oyston's study provides substantial food for thought and reflection. First and foremost, physicians, whatever their specialty, should be wary about providing email advice, especially to unknown "new" patients. The physician can have no way to accurately assess the patient under these circumstances. There will be no easily verifiable history and the physician will be completely dependent on the patient's story as it is related over the email. The physician will not have access to the many other senses and sources that support a legitimate differential diagnosis, such as a physical exam and the intuitive response to the patient's personal presentation of symptoms. As such, the physician is forming his opinion with information that is likely to be incomplete at best and inaccurate - or even untrue - at worst.

Even if the information gap can be minimized, there is considerable room for diagnostic error and uncertainly. Assume for instance that the email patient agreed to fax over her chart, as was requested by one of the physician subjects in Dr. Oyston's study. What guarantee does the physician have that this chart is authentic, complete, or contemporaneous? Would any physician treat solely on the written chart without interviewing and examining the patient personally, or at least verifying the authenticity of the material? Moreover, had the email patient forwarded the chart to the email anesthesiologist, this act would serve to bolster the impression that the physician had indeed undertaken to provide care, thus demonstrating that the physician did indeed assume a duty to care for the patient. In such a case, the assiduousness of the physician in seeking to review the chart would actually increase his exposure to liability.

This is not to say that email communication between physicians and patients is always risk-laden. In the situation where email is used to apprise the physician of the patient's progress, response to treatment, and well-being during an episode of care, ongoing email communication will benefit both physician and patient. However, in this context, the physician will have met and evaluated the patient, assuming the burden of care in the more traditional way. The physician will have a relationship with the patient, and be confident and knowledgeable about the patient's ability or limitations with respect to accurately describing signs and symptoms. Thus, the physician may proceed with greater assurance in advising the patient via email.

With respect to requests for advice and diagnosis over the Internet from "new" prospective patients, physicians would be well advised to proceed with the utmost caution. There is no duty to respond to an unsolicited message or plea for assistance. These email scenarios do not approximate "Good Samaritan" scenarios where a physician might reasonably believe there is a duty to provide assistance in an emergency. In fact, in the United States, "Good Samaritan" statutes provide qualified immunity for health care providers who have chosen to give unsolicited assistance at the scene of an accident. Generally the statute's definition of "scene of an accident" is narrow and would not include responding to a request for help through email. (One could argue that the duty relationship would be more substantiated if the patient has not independently solicited the physician's advice, but rather has responded to the physician's advertisement or offer of medical services extended to the general or a specific Internet audience, i.e. a disease-specific chat group.)

However, if the physician is unable to refrain from engaging in such a dialogue, he should be extremely circumspect in his responses, avoid engaging in differential diagnosis, and steer the patient to his or her own physician or an appropriate medical center [8]. In the final analysis, this is not just an issue of potential liability, but of the judicious practice of good medicine.
